# Alzheimer's disease - input of vitamin D with mEmantine assay (AD-IDEA trial): study protocol for a randomized controlled trial

**DOI:** 10.1186/1745-6215-12-230

**Published:** 2011-10-20

**Authors:** Cédric Annweiler, Bruno Fantino, Elsa Parot-Schinkel, Samuel Thiery, Jennifer Gautier, Olivier Beauchet

**Affiliations:** 1 Department of Neuroscience, Angers University Hospital; Angers University Memory Centre; UPRES EA 2646, University of Angers, UNAM, Angers, France; 2 Clinical Research Centre, Angers University Hospital, Angers, France

**Keywords:** Alzheimer's disease, vitamin D, memantine, clinical trial, older adults

## Abstract

**Background:**

Current treatments for Alzheimer's disease and related disorders (ADRD) are symptomatic and can only temporarily slow down ADRD. Future possibilities of care rely on multi-target drugs therapies that address simultaneously several pathophysiological processes leading to neurodegeneration. We hypothesized that the combination of memantine with vitamin D could be neuroprotective in ADRD, thereby limiting neuronal loss and cognitive decline. The aim of this trial is to compare the effect after 24 weeks of the oral intake of vitamin D_3 _(cholecalciferol) with the effect of a placebo on the change of cognitive performance in patients suffering from moderate ADRD and receiving memantine.

**Methods:**

The AD-IDEA Trial is a unicentre, double-blind, randomized, placebo-controlled, intent-to-treat, superiority trial. Patients aged 60 years and older presenting with moderate ADRD (i.e., Mini-Mental State Examination [MMSE] score between 10-20), hypovitaminosis D (i.e., serum 25-hydroxyvitamin D [25OHD] < 30 ng/mL), normocalcemia (i.e., serum calcium < 2.65 mmol/L) and receiving no antidementia treatment at time of inclusion are being recruited. All participants receive memantine 20 mg once daily -titrated in 5 mg increments over 4 weeks- and each one is randomized to one of the two treatment options: either cholecalciferol (one 100,000 IU drinking vial every 4 weeks) or placebo (administered at the same pace). One hundred and twenty participants are being recruited and treatment continues for 24 weeks. Primary outcome measure is change in cognitive performance using Alzheimer's Disease Assessment Scale-cognition score. Secondary outcomes are changes in other cognitive scores (MMSE, Frontal Assessment Battery, Trail Making Test parts A and B), change in functional performance (Activities of Daily Living scale, and 4-item Instrumental Activities of Daily Living scale), posture and gait (Timed Up & Go, Five Time Sit-to-Stand, spatio-temporal analysis of walking), as well as the between-groups comparison of compliance to treatment and tolerance. These outcomes are assessed at baseline, 12 and 24 weeks, together with the serum concentrations of 25OHD, calcium and parathyroid hormone.

**Discussion:**

The combination of memantine plus vitamin D may represent a new multi-target therapeutic class for the treatment of ADRD. The AD-IDEA Trial seeks to provide evidence on its efficacy in limiting cognitive and functional declines in ADRD.

**Trial Registration:**

ClinicalTrials.gov number, NCT01409694

## Background

Alzheimer's disease (AD) is the leading cause of dementia and loss of autonomy and independency in the elderly [1,2]. AD is characterized by a progressive decline of cognitive performance with a deleterious impact on social activities. The magnitude of this problem will increase over the next decades due to the demographic shift of the aging population. In order to mitigate and delay AD-related adverse effects, the development of new effective therapeutic strategies is essential [1,2].

In addition to a decrease in brain cholinergic activity that justifies the use of acetylcholinesterase inhibitors [1], AD is also marked by glutamatergic excitotoxicity [3,4] that results in neuronal death through two distinct mechanisms: immediate neuronal necrosis in the case of excessive intraneuronal calcium influx, and delayed neuronal apoptosis in the case of moderately excessive calcium influx with subsequent oxidative stress [5,6]. The clinical consequence of these chain reactions for glutamatergic neurons is the loss of learning and memory abilities characterizing dementia syndromes including AD and related disorders (ADRD) [1,3,4].

These pathophysiologic mechanisms explain why one of the most prescribed drugs to slow the progression of ADRD is memantine; a voltage-dependent, low-affinity, noncompetitive N-methyl-D-aspartate (NMDA) receptor antagonist [7,8]. The effectiveness of this drug was established in double-blind placebo-controlled trials in moderate to severe stages of dementia. Memantine has no immediate treatment effect, but after 3 to 6 months of use, patients report better cognitive performance and autonomy than those receiving placebo [7,8]. However, symptom improvement is transient and the memory scores then adopt a similar course to those in the placebo group [9]. Indeed, the use of memantine may result in the protection of glutamatergic neurons associated with the avoidance of neuronal necrosis phenomena subsequent to the excessive and prolonged influx of calcium into the cell, but AD patients using memantine are still exposed to oxidative stress and neuronal apoptosis. Thus, while NMDA receptor antagonists such as memantine allow symptomatic treatment of AD by temporarily slowing disease progression, they do not allow a preventive or curative treatment [7,8]. Coupling an antioxidant with memantine could therefore provide a solution to the problem of neuronal apoptosis induced by glutamatergic excitotoxicity.

Vitamin D is a neurosteroid hormone which crosses the blood-brain barrier and binds to vitamin D receptors (VDR) present in neurons and glial cells of the central nervous system including the hippocampus, the hypothalamus, the cortex and the subcortex [10-12]. More precisely, 1.25-dihydroxyvitamin D (active form of vitamin D) regulates the intra-neuronal calcium homeostasis via the regulation of voltage-gated calcium channels - thus preventing necrosis - [13], and has also exhibited neuroprotective properties against glutamate toxicity through antioxidant effects - thus preventing apoptosis. This antioxidant action was described in 2001 in cultures of rat mesencephalic cells [14]. This is particularly important in that over 70% of adults aged 75 and over are deficient in vitamin D [15,16] and should receive vitamin D supplementation, and especially since vitamin D deficiency has been associated with cognitive decline [10,17-20]. Thus, based on the neurosteroid properties of vitamin D, it can be argued that the correction of hypovitaminosis D may be protective against cognitive decline [11,20-23]. For illustration, it has already been shown that high exogenous supplies of vitamin D were associated with better cognitive performance compared to lower supplies [21,22]. What is more, it is interesting to note that the actions of vitamin D on the nervous system seem to be complementary and perfectly suited to those of memantine, and there is no reason to expect a deleterious interaction of these molecules [11].

Finally, the known actions of both these molecules are not reduced to learning and memory, but also improves physical performance. Indeed, each molecule taken separately has already been associated with an improvement of gait [24-26]. This is especially important since one of the main complication of ADRD relies on motor dysfunction illustrated by chronic gait disorders and loss of functional independence [2,27]. The expected improvement of cognitive performance in ADRD patients receiving both memantine and vitamin D could thus be accompanied by improved posture and gait performance.

We hypothesized that the combination of memantine plus vitamin D could be more protective in ADRD than taking memantine alone against the neuronal loss and the subsequent declines in cognitive and gait performance.

## Objectives

### Primary objective

The primary objective is to compare the effect after 24 weeks of the oral intake of vitamin D_3 _with the effect of a placebo on the evolution of cognitive performance in patients suffering from moderate ADRD and receiving memantine.

### Secondary objectives

The secondary objectives of the study are as follows:

- To compare the effect after 12 weeks of the oral intake of vitamin D_3 _with the effect of a placebo on the evolution of cognitive performance in patients suffering from moderate ADRD and receiving memantine.

- To compare the effect after 12 and 24 weeks of the oral intake of vitamin D_3 _with the effect of a placebo on the evolution of functional abilities in patients suffering from moderate ADRD and receiving memantine.

- To compare the effect after 12 and 24 weeks of the oral intake of vitamin D_3 _with the effect of a placebo on the evolution of postural and gait performance in patients suffering from moderate ADRD and receiving memantine.

- To determine the compliance to treatment and tolerancy of the oral intake of vitamin D_3 _in patients suffering from moderate ADRD and receiving memantine.

## Methods

### Design

**Figure 1:**
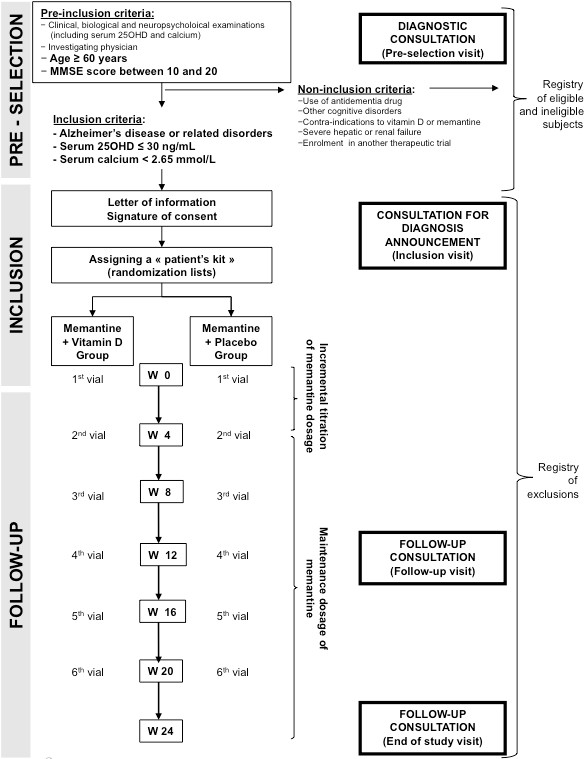
**Trial flow chart**. Flow chart of the Trial Study.

This is a unicentre, double-blind (with patient, carer, clinician, outcome assessor and investigators blinded), randomized, placebo-controlled, parallel group, intent-to-treat, superiority clinical trial. Figure 1 illustrates the trial design and Table 1 summarizes the timing of the trial. All participants receive trial interventions for 24 weeks as recommended by the European Medicines Agency (EMEA) guidelines [28]. The randomization is stratified depending on the nature of dementia (i.e., Alzheimer's disease [AD], or related disorders [RD]). Thus, for each stratum (2 strata), a distinct randomization list is established by the Clinical Research Centre (CRC) of Angers with the use of the N'Query randomization software. In order to best limit potential imbalances, a block randomization is used.

**Table 1:** Calendar summary

Period	Pre-selection	Inclusion	Follow-up	End of the study
**Week (W)**		**W0**	**W12**	**W24**

Pre-inclusion criteria	X			

Inclusion/Non-inclusion criteria		X		

Informed consent		X		

Inclusion clinical examination	X			

Analysis of posture and gait	X		X	X

Assigning a 'Patient's kit'according to inclusion number in compliance with randomization lists		X		

Serum 25OHD measurement	X		X	X

Serum calcium measurement	X		X	X

Serum parathyroid hormone measurement	X		X	X

Treatment administration		X		

Compliance (account of fully consumed vials)			X	X

Follow-up clinical examination			X	X

Cognitive scores (including ADAS-cog)	X		X	X

Functional scores	X		X	X

AEs and SAEs	X		X	X

Randomization lists were transmitted to the central pharmacy of Angers University Hospital which is in charge of assigning the 'patients' kits' in compliance with the randomization lists, and of shipping the kits containing containing all the medicines for the duration of the study (i.e., 322 tablets of memantine 10 mg, and 6 × 100,000 IU cholecalciferol or placebo vials) to the memory centre of Angers University Hospital. Of these 322 tablets of memantine, 316 are theoretically used during the 24-week follow-up, and an additional 6 tablets (equivalent to 3 days treatment) were added in each kit to be more flexible in determining the date of the final visit.

#### - 'Intervention' group

All participants start the treatment with memantine (Lundbeck Laboratory, Issy-les-Moulineaux, France) on the first day of the study. Memantine is administered according to the usual strategy, with upward titration of 5 mg per week during the first four weeks to reduce the risk of side effects. The final dosage is 20 mg per day, with no subsequent modification of dosage or specialty during the trial. In parallel, the participants of this group immediately commence vitamin D supplementation (cholecalciferol 100,000 IU [Crinex Laboratory, Montrouge, France], drinking solution, 2 mL vial) at a rate of 1 drinking vial of 100,000 IU cholecalciferol every month. In brief, the total dose is 600,000 IU over the study period of 168 days, starting with one vial at the time of inclusion, then at Week (W) 4, W8, W12, W16 and W20. It is generally recognized that each additional 100 IU of vitamin D per day raises serum 25OHD concentration by 0.7 ng/mL [29]. The daily dose of 3,571 IU in the AD-IDEA trial is thus expected to raise the serum 25OHD concentration by at least 25 ng/mL, with a concentration ultimately reached > 30 ng/mL. This is all the more important in that, based on the knowledge of vitamin D effects in organs other than brain, it seems that raising serum 25OHD concentrations above 30 ng/mL is necessary to obtain an effect on non-skeletal targets [30]. We assume that the 24 week assessment will capture a treatment effect since vitamin D supplementation > 6 months is not more effective than shorter treatment ≤ 6 months for the prevention of non-skeletal adverse events [31]. Finally, it has to be quoted that the chosen dose of 3,571 IU per day does not reach toxic doses (i.e., 10,000 IU per day) and raises vitamin D concentrations within non-toxic limits (i.e., 150 ng/mL) [32]. As a consequence, the dose of vitamin D supplementation will not be adjusted except in the case of a serious adverse event such as hypercalcemia, possibly resulting from the intestinal absorption of dietary calcium under the action of vitamin D [16,30]. In this case, vitamin D supplementation is stopped and the participant is released prematurely from the study.

#### - 'Placebo' group

Participants in this arm start the treatment with memantine at the same rate as the 'Intervention' group. They also immediately start cholecalciferol placebo (Placebo [Crinex Laboratory, Montrouge, France], drinking solution, 2 mL vial) administered at the same pace (i.e., W0, W4, W8, W12, W16 and W20). The placebo drinking solution contains all the excipients present in the cholecalciferol vial and is not distinguishable from the active product (vitamin D).

Regardless of groups, enrolled participants are also provided with oral and written information that they cannot, in any case, be prescribed medication containing vitamin D or memantine or acetylcholinesterase inhibitors outside of the trial for the duration of the study.

### Planned eligibility criteria

#### Pre-inclusion criteria

People are considered for possible inclusion if they are aged 60 years and older, meet the DMS-IV criteria for dementia [33] at a moderate stage (MMSE score between 10 and 20 inclusively) [34] and are affiliated to French Social Security.

#### Inclusion criteria

People are eligible to participate if they meet the National Institute of Neurological and Communicative Disorders and Stroke/Alzheimer's Disease and Related Disorders Association (NINCDS/ADRDA) criteria [35], have hypovitaminosis D (i.e., serum 25-hydroxyvitamin D [25OHD] concentration ≤30 ng/mL) [36] and no hypercalcemia (i.e., serum calcium concentration < 2,65 mmol/L). Participants must also have given and signed an informed consent form to participate in the trial (or informed consent form obtained from the trusted person or legal representative, as appropriate).

#### Non-inclusion criteria

The following non-inclusion criteria are considered: severe, unstable or poorly controlled medical conditions apparent from physical examination or clinical history, severe hepatic or renal failure, the use of standard antidementia drugs (i.e., acetylcholinesterase inhibitors, memantine, or vasodilatators) in the past 60 days, other cognitive disorders (untreated dysthyroidy, deficiency in vitamin B9 or B12, alcohol-related dementia, history of syphilis or of strategic stroke, delirium at time of inclusion [Confusion Assessment Method positive] [37], presence of severe depressive symptoms [15-item Geriatric Depression Scale score ≥ 10]) [38], contra-indications to memantine or vitamin D including granulomatosis, and enrolment in another simultaneous clinical trial.

### Recruitment/consent procedures

Participants are identified from patients with ADRD meeting study pre-inclusion criteria who are being followed up in Angers University Memory Centre and other components of specialist mental health, geriatric medicine or neurology services. Once a potential participant is identified and meets the eligibility criteria, the investigating physician provides the patient (as well as a family member, a trusted person or a legal representative, as appropriate) written and oral information on the study in an understandable language, and obtains written consent to take part in the study in line with the trial standard operating procedures. When possible, fully informed consent is obtained from the patient. When ADRD patient is unable to give fully informed consent, agreement to participate in the study is obtained from the trusted person or from the legal representative, as appropriate, and the patient is not enrolled if s/he refuses or shows significant distress. If neither is available to consent, the patient is ineligible for the study. In case of non-inclusion (if the patient does not meet the above selection criteria) or refusal to participate in the study, the investigating physician will record the existence or absence of selection criteria and the causes of non-participation in the study, and copy them in the "Registry of non-eligible and eligible patients".

### Assessments

Study assessment measures will be applied at baseline prior to randomization, at W12 and at W24. All measures in the study are already carried out systematically in every patient admitted to Angers University Memory Centre. Experience has shown that this mode of operation is feasible and appreciated by patients and their families, and has never caused a breakdown in care so far. For this reason, the large number of measures is not expected to exacerbate the loss to follow-up. To ensure adherence to the intervention, nurses are prescribed for all participants to administer medications safely and effectively. In addition, phone calls are planned to keep in touch with the participant and to recall the date of follow-up visits. Trial completion is defined as completion of 24 weeks on the trial medication or discontinuation of follow-up for any cause. Participants who discontinue taking the trial medications are encouraged to remain in follow-up. Arrangements for continued provision of the trial medication at the end of the trial is made on an individual basis thanks to a centralized serum 25OHD measurement at W24 which allows the investigating physician in charge of the patient making rapid and appropriate decisions regarding carrying on, modifying or discontinuing vitamin D supplementation at the end of the study.

#### Primary Outcome Measure

##### - Alzheimer's Disease Assessment Scale-cognition (ADAS-cog) score

The ADAS-cog [39] is a well-established measure of cognitive function in older adults. It shows good test-retest and inter-rater reliability and performs satisfactorily against more detailed measures of cognitive function. Score range varies from 0 to 70. The more mistakes, the higher the score is. A normal score is defined as a score lower than 10. A 70-point score is a sign of severe dementia. A variation of at least 3 points during the study is considered as clinically relevant [40].

#### Secondary Outcomes Measures

##### - Mini-Mental State Examination (MMSE) score

The MMSE [41] is used to assess cognitive funtions and mnesic abilities. The test consists in a series of 30 questions composed of five sections (orientation, registration, attention-calculation, recall, and language), with final score graded out of 30 points. A final score below or equal to 25 points corresponds to a dementia diagnosis. A score between 25 and 21 indicates mild dementia. A score between 20 and 10 indicates moderate dementia. A score below 10 indicates severe dementia [34]. A variation of 2 points during the study is considered as clinically relevant [42].

##### - Frontal Assessment Battery (FAB) score

The FAB explores executive functions [43]. It consists of 6 subtests marked on 3 points with a total score of 18. A score under 14 is abnormal [43]. The matching task explores conceptual elaboration; the verbal fluency task explores lexical evocation and mental shifting; the search for prehension behavior explores environmental autonomy; Luria's motor sequences explore programming abilities; the conflicting instructions tasks explore sensitivity to interference; finally, the Go/No Go task explores motor inhibition.

##### - Trail Making Test (TMT) parts A and B score

The TMT [44] assesses mental shifting and consists of two parts. First, the subject is required to draw lines to connect numbers in ascending order as quickly as possible. This part (TMT A) assesses visual perception rapidity and psychomotor rapidity. The second part (TMT B) assesses mental shifting and the subject's attention ability since s/he is required to do the same as for TMT A, but alternating between numbers and letters. The subject is asked to perform the task as quickly as possible without lifting his/her pen. If the experimentor sees a mistake, s/he tells the patient. The following items are recorded: time spent (part A, part B; difference between time spent for parts A and B), and the number of errors (total number of errors parts A and B; perseverant errors for part B; difference in number of errors in parts A and B). If the time to complete TMT B is longer than 240 seconds, then the test is stopped and the number of figures connected in the allotted time as well as the number of errors is noted.

##### - Activities of Daily Living scale (ADL) score

The patient's ability to perform the main activities of daily living and to adapt to his/her environment is measured thanks to this systemized questionnaire exploring activities of body care, dressing, elimination and continence, mobility and eating [45]. The ability to perform an item is worth 1 point. A maximum score of 6 out of 6 corresponds to a state of independence. A score lower than 3 out of 6 defines a state of dependence.

##### - 4-item Instrumental Activities of Daily Living scale (IADL-PAQUID) score

The purpose of this screening test is to assess behaviors and the use of common tools: using transportation, managing finances, using the phone, managing medicines [46]. If one of these functions is affected, it is marked 1. Disability starts from a score of 2 out of 4.

##### - Performed Timed "Up & Go" (TUG) and Imagined Timed "Up & Go" (iTUG)

The TUG assesses gait and postural performance. The TUG is to measure time in seconds used by a subject to rise from his chair, walk three meters, turn around and return to the sitting position [47]. A time longer than 20 seconds is pathological. The iTUG assesses motor imagery. It is to make TUG first, and then to sit on the chair and imagine doing it again [48]. The time taken by the subject to imagine the iTUG is measured in seconds. Age and cognitive decline are associated with significantly increased TUG and decreased iTUG [48].

##### - Five Time Sit-to-Stand test (FTSS)

The FTSS measure patient's postural abilities. The test is to stand up from a chair five times as quickly as possible without pushing off. Performance is measured with a stopwatch in seconds, as the time from the initial seated position to the final seated position after completing five stands. A time longer than 15 seconds is abnormal and is associated with physical and cognitive impairments [49].

##### - Spatio-temporal analysis of walking

These parameters are measured using GAITRite^® ^system (972 cm long, active electronic surface area 792 × 610 cm, with a total of 29,952 pressure sensors, scanning frequency 60 Hz, software version 3.8, CIR System, Havertown, PA). Participants walk in a quiet, well-lit room wearing their own footwear according to European guidelines for spatio-temporal gait analysis in older adults [50]. The walk tests are performed consecutively with and without a concurrent attentional task [27].

##### - *Compliance*

Empty, full or partially consumed vials and tablets of memantine are stored by the subjects, brought back to the investigating physician during each follow-up consultation, and are counted in order to measure compliance. In addition, the change of serum 25OHD concentration (including the correction of hypovitaminosis D) will validate a posteriori the actual treatment intake within the 'Intervention' group.

##### - *Blood samples collection procedures*

All subjects are tested for 25OHD concentration at W0, and actually included subjects are also tested at W12 and W24. Blood sampling are done by a clinical research nurse at each consultation. Serum 25OHD concentration is then measured with the DiaSorin radioimmunoassay (RIA) kit (DiaSorin, Stillwater, MN, USA) locally at the University Hospital at Angers, France, to homogenize the measuring technique. The RIA DiaSorin kit is the most used kit for such studies and recognizes both D_2 _and D_3_. With this method, there is no interference of lipids, which is often observed in other non chromatographic assays of 25OHD. Additionally, this method correlates with the reference method (high-performance liquid chromatography with mass spectrometry [HPLC-MS]). The intra- and interassay precisions are respectively 5.2% and 11.3% (range in normal adults aged 20-60 yr, 30-125 ng/mL).

##### - Safety parameters

The safety assessment parameters are:

- Clinical: delirium, somnolence, asthenia, vertigo, headache, epilepsy, nausea and vomiting, heart failure, lumbar or right hypochondre pain in the case of biliary or renal colics revealing calcic lithiasis (objectivized with echography in the case of clinical suspicion), thrombosis (objectivized with doppler in the case of clinical suspicion);

- Biological: serum calcium concentration. The serum concentration of calcium is measured at baseline assessment, then at W12 and W24 to monitor the occurrence of hypercalcemia > 2,65 mmol/L [32]. The occurrence of hypercalcemia leads to double-blind termination and premature withdrawal from the study, stops the vitamin D supplementation and potential calcium supplements, and triggers further complementary non-specific examinations to find the cause of hypercalcemia for which vitamin D intake could not be held responsible at first [16].

### Statistics

#### Sample Size Calculation

The trial aims to recruit 120 patients over a period of 52 weeks. According to previous literature, we estimate that the change in ADAS-cog score after 24 weeks in patients receiving memantine will be 1.25 ± 5 points on average [8]. In addition, it is usually considered in clinical trials among AD patients that the minimum clinically relevant change in ADAS-cog score is at least 3 points [40].

Thus, assuming a reference value of 1 and a variance of 25 (standard deviation estimated at 5), a two-sided test with an alpha risk of 5%, and a power (1 - beta) of 99%, the sample size to detect a 3-point difference in change from baseline in the ADAS-cog between the 'Intervention' group and the 'Placebo' group, is 52 subjects per group (total 104 subjects).

Taking into account the loss to follow-up (estimated at 15%), it is necessary to include a total of 120 subjects (60 per group). This sample size has also adequate power (i.e., 90%) to detect a smaller difference in ADAS-cog score (i.e., 2 points). The sample size calculation also assumes accounting for up to 12 covariables anticipating multivariate analyses, including the change in comorbid conditions possibly due to vitamin D supplementation [51] with subsequent indirect cognitive benefits (using the Cumulative Illness Rating Scale for Geriatrics [CIRS-G] score) [52], the education level [1], the season tested [16], and the serum concentrations of parathyroid hormone (PTH) and calcium [53].

#### Analyses

The effect of vitamin D supplementation compared to placebo will be determined using evaluation criteria, which are the changes in cognitive, functional and physical scores between W0 and W12 and between W0 and W24, using independent samples t-test or Mann-Whitney U test; and the between-group comparisons of changes in cognitive, functional and physical scores between W0 and W12, and between W0 and W24, using an analysis of covariance.

Any exploratory analyses here will use multivariate logistic and proportional hazards regression. In addition, the stratification criterion for randomization (i.e., the nature of dementia: AD or RD) will be also taken into account in the multivariate models. Excessive subgroup analyses can give rise to misleading results and therefore all subgroup investigations will be interpreted cautiously. In particular, subgroup analysis will be conducted depending on the severity of hypovitaminosis D at the beginning of the study (i.e., serum 25OHD concentration below or above 10 ng/mL) and on the severity of dementia at the beginning of the study (i.e., MMSE score below or above 15). Finally, special attention will be paid to participants that, while having hypovitaminosis D at the beginning, did not show high levels of PTH, to determine whether this group differentiates of the PTH responders in terms of therapeutic benefits.

### Ethical considerations

The protocol received Angers Research Ethics Committee approval, and was approved by the AFSSAPS (French health products safety agency). The trial is conducted in compliance with the European Union Clinical Trials Directive (2001/20/EC), the French National Commission for Information Technology and Freedom (1978), the Public Health Code of Ethics, the International Conference on Harmonization guidelines for Good Clinical Practice (CPMP/ICH/135/95), the principles of the Declaration of Helsinki (1996) and other requirements as appropriate.

An Independent Data Monitoring Committee (IDMC) will monitor the progress of the trial including: recruitment, protocol adherence, serious adverse events and side effects of treatment as well as the difference between the trial treatments on the primary outcome measures. The IDMC will produce a report to the Trial Steering Committee (TSC) after every meeting and can recommend premature closure of the trial following clear evidence of benefit or harm in accordance with the IDMC charter.

The main ethical issue here is related to the delay of vitamin D supplementation in the 'Placebo' group. This treatment delay of 24 weeks seems yet acceptable since vitamin D supplementation has not yet proved efficient for ADRD patients. Due to this reasonable doubt and to the lack of standard treatments to be associated with memantine, resorting to a placebo is thus conceivable and justifies the study.

In addition, subject participating in this research can also expect personal benefits. Individual expected benefits are:

- All included subjects receive memantine, a standard antidementia treatment that is appropriate for moderate ADRD.

- For subjects within the 'Intervention' group, the treatment will correct the hypovitaminosis D.

- Since presenting with hypovitaminosis D is an inclusion criterion, participating in this study constitutes an opportunity even for subjects receiving a placebo. Indeed, in the hypothesis of an effectiveness of vitamin D, the subjects in the 'Placebo' group would undergo a potential transient loss of opportunity (i.e., being deficient and receiving a placebo during the study period) balanced by permanent growing opportunities at the end of the study (i.e., receiving vitamin D supplementation).

- The expected benefits for the 'Intervention' group are potentially important because, in the hypothesis of an efficient action of the combination of memantine plus vitamin D on brain, participation in this research may prevent cognitive decline and improve ADRD symptoms.

The expected collective benefits for this research are an improvement of the knowledge of the non-skeletal effects of vitamin D, and in particular of the cognitive effects of the combination of vitamin D plus memantine.

## Discussion

To date there is no curative treatment for ADRD and existing symptomatic treatments only have a transient efficacy.

The novel idea of combining memantine and vitamin D is based on the supposed complementarity of their actions. In particular, it appears that cognitive declines in AD and hypovitaminosis D have a partially common pathophysiological pathway based on the calcium neurotoxicity and the alteration of protective mechanisms against glutamatergic excitotoxicity [11,20]. The use of vitamin D in ADRD patients seems thus justified, particularly among the deficient ones who are at least 70% of patients visiting memory centres [15].

In addition to its neurosteroid "multi-target" effects, testing vitamin D therapy sounds interesting in that vitamin D can be associated with current antidementia drugs as part of a "multi-drug" regimen [23]. This is an important point when considering randomized controlled trials since it seems almost impossible to get a trial approved to examine the effectiveness of vitamin D alone in ADRD patients after having removed standard therapies. In addition, conducting a placebo-controlled clinical trial in subjects with overt vitamin D deficiency for a 6-month period is acceptable because of the absence of expected accident linked to vitamin D deficiency within this short period [23,54]. Despite all these facilitating arguments and the growing interest in non-skeletal effects of vitamin D [30], the AD-IDEA Trial is, from the best of our knowledge, the first randomized placebo-controlled trial on the effectiveness of vitamin D in ADRD patients [23]. Its randomized double-blind placebo-controlled design minimises the risk of bias. Yet, generalization of the AD-IDEA findings will be limited by the single-centre design. The choice to conduct the trial in one single memory centre was explained by considerations of feasibility. If proven, the finding of an effective response would prompt to extend the trial on a variety of memory centers in several international hospitals, with studied samples not restricted to senior patients with moderate stages of dementia. Also, conducting longer-term follow-up of 12 months and more would provide additional information on the persistence of the effect [28].

The combination of memantine plus vitamin D may represent a new multi-target approach for ADRD. The results of the AD-IDEA Trial will make a substantial contribution to this new orientation of research, and in all probability will provide clear evidence on the efficacy of this new pharmaceutical composition in limiting cognitive and functional declines in ADRD.

## Trial status

Recruiting

## Competing interests

The authors declare no conflicts of interest. They have no personal relevant financial interest in this manuscript.

## Authors' contributions

CA conceived the study, assembled the group of co-investigators and, with them, developed and finalised the protocol. All of the above have set up the trial site and made it ready to perform the trial. CA, EPS and OB provided statistical advice in the design of the study and its on-going evolution. CA, OB, EPS, ST and JG managed the trial data management and coordinated development of the trial. CA, OB and BF particularly developed this publication describing the trial protocol. All the authors read and approved the final manuscript.
